# Impact of gender on contractile function in human skinned fibers in condition of volume-overload

**DOI:** 10.1186/1749-8090-10-S1-A257

**Published:** 2015-12-16

**Authors:** Constanze Bening, Nicole Stumpf, Christian-friedrich Vahl

**Affiliations:** 1 Department of Cardiothoracic -and Vascular Surgery, University Hospital Mainz, 55131 Mainz, Germany

## Background/Introduction

Gender-based differences in the cardiac morphology and function in patients with volume-overload cardiac pathologies has been widely examined. However the relationship between gender and contractility is not well defined.

## Aims/Objectives

Therefore we evaluate the gender influence on contractile capacity in patients with volume overload, present with severe mitral valve regurgitation and preserved left ventricular function.

## Method

Right auricle samples from 40 patients (20 male patients, 67 ± 9 years; 20 female patients, 68 ± 9 years) with severe mitral regurgitation, scheduled for elective mitral valve surgery, were obtained before extracorporal circulation (ECC). The fibers were prepared and skinned to remove membrane-dependent properties and exposed to gradual increase of calcium concentration.

## Results

Female fibers achieve higher force values than male patients at the highest step of calcium concentration (pCa 4.0: 3.9 ± 0.5 mN versus 2.9 ± 0.9 mN, p 0.02). Male and female fibers show an anticyclical course when exposed to increasing steps of calcium concentrations: starting with lower force values at lower calcium concentrations in males, the steepness of pCa-force-curve is flatter compared to female counterparts (pCa 6.5 males: 1.3 ± 0.3 mN versus females 0.203 ± 0.1 mN, p 0.03 and pCa 6.0 males: 1.5 ± 0.4 mN versus females 0.3 ± 0.09 mN, p 0.09). At higher force values females achieve higher maximal forces and lower force values for males (pCa 4.0 females 3.9 ± 0.5mN versus males 2.9 ± 0.9 mN, p 0.02; and pCa 4.5 females 2.83 ± 0.3 mN versus 2.1 ± 0.1 mN, p 0.02).

Calcium sensitivity, given as half maximal concentration, is achieved at pCa 5.0 in females and between 6 and 5.5 for males (p 0.04).

## Discussion/Conclusion

Male and female fibers from patients with severe mitral regurgitation show adverse calcium induced force properties. Male fibers achieve lower maximal force values, starting at higher base values in low calcium concentration condition and might therefore have higher sensitivity to calcium.

